# Organ Cultures for Retinal Diseases

**DOI:** 10.3389/fnins.2020.583392

**Published:** 2020-11-25

**Authors:** José Hurst, Agnes Fietz, Teresa Tsai, Stephanie C. Joachim, Sven Schnichels

**Affiliations:** ^1^Center for Ophthalmology, University Eye Hospital, University of Tübingen, Tübingen, Germany; ^2^Experimental Eye Research Institute, University Eye Hospital, Ruhr-University Bochum, Bochum, Germany

**Keywords:** *ex vivo*, retinal organ culture, age-related macular degeneration, glaucoma, retinitis pigmentosa, central artery occlusion

## Abstract

The successful development of novel therapies is closely linked with understanding the underlying pathomechanisms of a disease. To do so, model systems that reflect human diseases and allow for the evaluation of new therapeutic approaches are needed. Yet, preclinical animal studies often have limited success in predicting human physiology, pathology, and therapeutic responses. Moreover, animal testing is facing increasing ethical and bureaucratic hurdles, while human cell cultures are limited in their ability to represent *in vivo* situations due to the lack of the tissue microenvironment, which may alter cellular responses. To overcome these struggles, organ cultures, especially those of complex organs such as the retina, can be used to study physiological reactions to substances or stressors. Human and animal organ cultures are now well established and recognized. This mini-review discusses how retinal organ cultures can be used to preserve tissue architecture more realistically and therefore better represent disease-related changes. It also shows how molecular biological, biochemical, and histological techniques can be combined to investigate how anatomical localization may alter cellular responses. Examples for the use of retinal organ cultures, including models to study age-related macular degeneration (AMD), *retinitis pigmentosa* (RP), central artery occlusion (CRAO), and glaucoma are presented, and their advantages and disadvantages are discussed. We conclude that organ cultures significantly improve our understanding of complex retinal diseases and may advance treatment testing without the need for animal testing.

## Introduction

Cell cultures have become indispensable in translational research. Many preclinical evaluations, like toxicity testing or drug screening, are still based on standardized cell lines. Usually, these assays are simple, inexpensive, reproducible, and allow a fast and efficient screening for cell proliferation, metabolism, viability, and apoptosis (Schnichels et al., 2012a, 2013; Hurst et al., 2017b). Cell cultures are the method of choice to observe the reaction of a specific cell type. Already 20 years ago, it was published that at least 15–20% of all immortalized cell lines used in research are contaminated or wrongly categorized, resulting in non-transferable data (MacLeod et al., 1999). Moreover, immortalized cells react to stimuli differently than primary cells (MacLeod et al., 1999), and for several cell types, no corresponding cell lines are available. For example, primary photoreceptor cells are very difficult to cultivate under *in vitro* conditions and degenerate rapidly after dissociation from the retina (Fontaine et al., 1998; Romano and Hicks, 2007). The same applies to retinal ganglion cells (RGCs). The only existing RGC line is the presumably rat-derived RGC-5 cell line, which was mainly used for glaucoma research. Over the years, the literature has increasingly described altered properties of these cells, showing a reduced expression of specific neural and ganglion cell markers (Harper et al., 2009; Van Bergen et al., 2009; Ganapathy et al., 2010; Wood et al., 2010). In the meantime, early contamination with a murine cell line was assumed (Krishnamoorthy et al., 2013). Until today, the origin of the RGC-5 cell line could not be verified, and researchers are strongly advised to avoid using it (Clark et al., 2013; Krishnamoorthy et al., 2013; Sippl and Tamm, 2014).

In general, cell cultures do not mimic the tissue homeostasis, physiology, and interactions that occur between different cell types within a tissue *in vivo*; thus, *in vitro* models can never completely replace *in vivo* ones. However, *in vivo* experiments also have their disadvantages: They are rather expensive and time-consuming. Even after long-term and well-performed research, not a single treatment option for sporadic neurodegenerative diseases has proven its value in registration-sized clinical trials (Li et al., 2018; Murali et al., 2019).

In response to these gaps and struggles, organ cultures are gaining ground, including organoids generated from stem cells, tissue parts taken from organs, and construction of new 3D structures with different cell types, containing cell lines, primary or stem cells depending on the respective research approach (Aizawa and Shoichet, 2012; Hunt et al., 2017). The big advantage of organ cultures is that elementary structures of the organ are modeled, allowing investigations of complex interactions.

This especially holds for ophthalmology, where *ex vivo* models of the cornea and retina are common research tools with the additional positive effect of reducing the number of animals used in research and, most importantly, replacing the need for certain *in vivo* procedures (Schnichels et al., 2019).

## Organ Cultures: Advantages and Challenges

Nowadays, numerous retinal organ models from different mammalian species are well established. However, compared to the cultivation of cornea models, retinal organ cultures are more difficult to maintain, mainly due to the different cell types involved, which are generally more complex to cultivate. Especially designed growth medium, containing growth factors for neurons, supports cell survival. Co-cultivation with retinal pigment epithelium (RPE) cells improves tissue structure, cellular organization, and preservation of photoreceptors (Di Lauro et al., 2016; Schnichels et al., 2020). Zhao and Barnstable (Zhao and Barnstable, 1996) pointed out that retinoic acid but not basic fibroblast growth factor (bFGF) promotes rod photoreceptor differentiation, whereas bFGF endorses RGC differentiation in *ex vivo* rat retinae. B-27 and N2 are the most commonly used supplements in retinal explant culture; although they were reported to be useful in rat retinal culture, the same benefit is not necessarily valid for human retinal explants (Osborne et al., 2016; Li et al., 2018; Murali et al., 2019).

To establish a functional retinal model, several challenges need to be overcome. A major problem is the limited viability of most retinal organ culture systems due to axotomy or lack of blood flow and biomechanical tissue support leading to metabolic disorders and cell death (Åkerström et al., 2017; Schnichels et al., 2019). This mainly affects RGCs, but also other cell types such as macroglia, displaying gliosis (Tanihara et al., 1997; Wurm et al., 2011; Joachim et al., 2012; Hurst et al., 2017a) and reducing the time frame for experiments with these cultures.

Likewise, depending on the assay, small effects of individual cell types are lost in the total reaction or very heterogeneous results are obtained. Biological diversity might be an issue; heterogeneity and variability between samples also occur. This can be reduced if retinae from genetically identical laboratory animals, such as mice or rats, of the same age and sex are used. Likewise, an altered expression profile may occur due to the preparation, cultivation, or medium composition (Roark et al., 1992; Fernandez-Bueno et al., 2012; Schnichels et al., 2019). This deviation from the *in vivo* situation should always be taken into account when results from an *ex vivo* approach are interpreted.

Due to their matching anatomical and physiological structures, human retinal organ cultures represent the ideal model system. *Ex vivo* human cultures can be cultivated up to 6 weeks, as demonstrated by Engelsberg et al. (Engelsberg et al., 2008) on human fetal retinal explants. Osborne et al. (Osborne et al., 2016) showed that neither donor age nor postmortem time significantly affects the initial expression levels of RGC markers and that donor retinae are suitable as a long-term model for RGC degeneration. Nevertheless, the availability of human organs is extremely low and severe ethical issues arise when using fetal eyes. One solution is the use of organoids produced from stem cells or even from patient cells, which mimic retinal structure and composition, but they also have their disadvantages (Eiraku et al., 2011). Therefore, the aim is to establish retinal organ cultures from species reflecting human morphology and physiology very closely.

The most commonly used animals in ophthalmologic research, rodent, dog, cow, and pig retinae do not have a macula but instead possess a region called *area centralis*. Furthermore, instead of three different cone types as humans, lower mammals only have two types (Peichl, 2005; Murali et al., 2019). As a result of these anatomical differences, the development of some diseases is different.

Nevertheless, the use of porcine or bovine retinae has several advantages. During research studies on RGCs, porcine retinal explants have been shown to remain viable for 8 days (Kuehn et al., 2016, 2017; Hurst et al., 2017a). Not to mention the fact that no laboratory animals have to be bred and killed. Those animals have larger eyes, so several explants can be obtained from a single eye, leading to more reliable data. The anatomy and vascularization of porcine eye cultures resemble those of the human eye (Guduric-Fuchs et al., 2009).

Taken together, advantages of *ex vivo* organ cultures are multiple: numbers of laboratory animals can be reduced, the experiments are less cost intensive, larger sample quantities can be generated, and there is better standardization of the experiment. In addition, the application of possible therapeutics is easier than that *in vivo* (Schnichels et al., 2019) and different retinal cell types can be studied under the same condition (Hurst et al., 2017a).

## Experimental Methods on Organ Cultures

The further development of certain techniques contributes to the development of more useful organ cultures for ophthalmologic research. For example, new methods for the introduction of DNA/vectors/plasmids are available or under development, which overcome the problem of uneven penetration or cellular uptake, as well as vector transcription in, e.g., neuronal cells (Marwick and Hardingham, 2017; Jüttner et al., 2019). Besides using viral vectors for tissue transfection, this includes magnetofection on *ex vivo* organ cultures (Yang et al., 2008; Soto-Sanchez et al., 2015; Jüttner et al., 2019). In order to regulate the expression of the vectors, new promoters were invented, which can be read in the retina under non-mitotic conditions (Dalkara et al., 2013, 2016).

**FIGURE 1:**
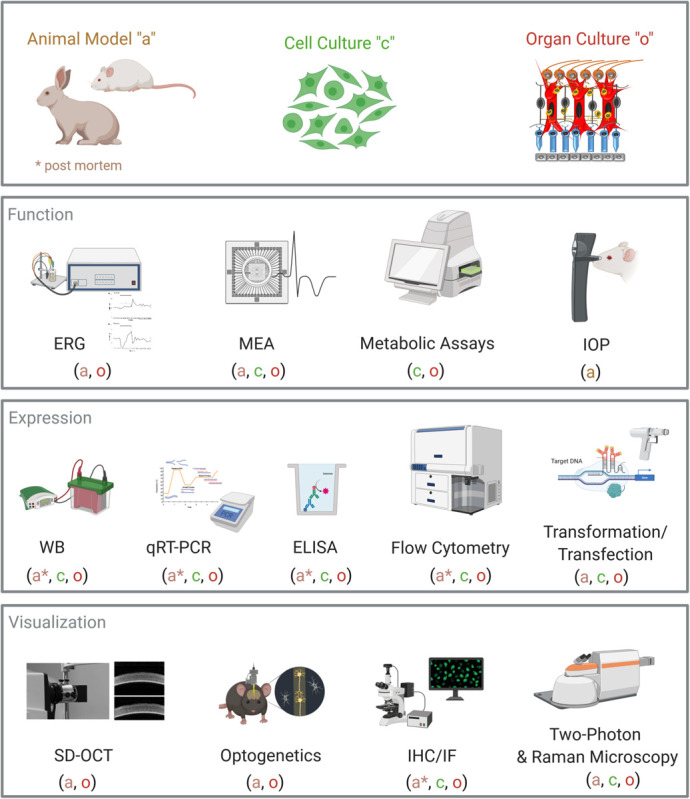
Overview of analytical methods for retinal organ cultures. Common laboratory and diagnostic methods are displayed. For each method, it is noted if it can be performed on animal models (a), on cell cultures (c), or on organ cultures (o). Abbreviations: ERG, electroretinogram; MEA, microelectrode array; IOP, intraocular pressure; WB, Western blot; qRT-PCR, quantitative real-time PCR; ELISA, enzyme-linked immunosorbent assay; SD-OCT, spectral domain optical coherence tomography; IHC, immunohistochemistry; IF, immunofluorescence. Created with BioRender.com.

There are numerous bioassays to investigate cell metabolism, cell death, or proliferation. Many of these assays can also be performed on retinal tissue, such as the measurement of reactive oxygen species (ROS) production from hypoxia-stressed retinal explants (Maliha et al., 2019; Sasaki et al., 2019; Figure 1).

Furthermore, more sophisticated techniques like single-cell Western blot or single-cell PCR allow the analysis of individual cells in tissues (Hughes et al., 2014). Using non-linear optical microscopy, in particular confocal Raman microscopy or two-photon-excited fluorescence microscopy, limitations can be overcome, providing depth penetration up to 1 mm without destroying the tissue (Helmchen and Denk, 2005; Sharma et al., 2016; Gomes da Costa et al., 2019). In addition to the common laboratory methods, specialized methods from neurobiology and ophthalmology can be applied to retinal organ cultures (Figure 1).

### Optical Coherence Tomography

Optical coherence tomography (OCT) is a well-established diagnostic imaging technique that allows both qualitative (morphology) and quantitative (thickness) analyses of the retinal architecture (Fujimoto et al., 1998). OCT examination of the retina is used to diagnose retinal diseases and measure therapeutic effects. Since OCT has become available, correlations between anatomy on OCT and visual function have been investigated in a number of retinal diseases (Keane et al., 2008). Using a specially developed holder, OCT examinations can be performed *ex vivo* on retinal organ cultures (Schnichels et al., 2016). On the one hand, OCT is a good alternative if you do not have access to confocal or multiphoton microscopy; on the other hand, OCT measurements allow the comparison of experimental data with patient data, can be repeated several times, and are a simple and fast method that does not require fixation of the retina.

### Electroretinograms

Electroretinograms (ERGs) record the electrical responses of various retinal cell types, including photoreceptors and inner retinal cells (bipolar and amacrine cells). ERG recordings in isolated retinae are highly standardized in different species like human, bovine, or retinal tissue deriving from rodents (Kuchler et al., 1956; Albanna et al., 2009; Hurst et al., 2018). In contrast to *in vivo* experiments, *ex vivo* retinae are not affected by the depth of anesthesia and electrode placement (Homma et al., 2009). ERG is used in the clinic to diagnose various retinal diseases such as RP, choroideremia, or achromatopsia. It serves as a sensitive pharmacological tool to test effects of applied substances on photoreceptors and neurons that contribute to the generation of the a- and b-wave (Januschowski et al., 2014a,b). Luke et al. (2009) investigated the effects of ranibizumab (Lucentis) on retinal function in *ex vivo* bovine retinae. Ranibizumab blocks vascular endothelial growth factor (VEGF), the major factor in age-related macular degeneration (AMD) progress. Ranibizumab therapy could induce retinal dysfunction; however, no reduction of the a- or b-wave in the ERG was observed (Luke et al., 2009).

### Microelectrode Array Recording

Microelectrode array (MEA) recording is the method of choice to measure electrical activity in cell and tissue preparations. MEAs were developed on cell cultures and are now used *ex vivo* in tissue for pharmacological studies and *in vivo* as implantable devices in brain pacemakers or cochlear implants (Obien et al., 2014). They consist of a multitude of microscopic electrodes on the surface of a glass plate on which the cells are cultivated. The electrodes enable electrical stimulation and simultaneous measurement of electrical activity of ganglion cells. This allows for accurate mapping of RGCs in neuronal tissue and to use the gained information for disease models (Baden et al., 2016; Zeck, 2018; Ran et al., 2020). In addition, MEAs are also ideal biosensors for measuring acute and chronic effects of drugs and toxins (Rosolen et al., 2008; Tao et al., 2020).

## Disease Models and Therapy Tests on Retinal Organ Cultures

Regrettably, for certain retinal disorders like AMD, glaucoma, RP, diabetic retinopathy, or CRAO, relevant *in vivo* models are missing or are imperfect for studying underlying pathophysiology. However, there is a high number of retinal organ cultures of different species with which a spectrum of disease simulations can be performed. These models are used to visualize the cellular basis of retinal pathogenesis to elucidate the role of specific genes and proteins in normal and disease processes and to test therapies without the use of laboratory animals. For example, Huang et al. (2017) explored new methods such as injection by ultrasound for retinal drug delivery. Intravitreal injection is the most common route of administration, and the vitreous body and the inner limiting membrane are the main obstacles to efficient administration. For this experiment, bovine retina with vitreous was cultured to preserve the membranes and simulate the hindrances (Huang et al., 2017). In the following, some published models are presented to get an impression of the potential of retinal organ cultures.

### Age-Related Macular Degeneration

AMD represents a complex and heterogeneous disease that affects the macula, with both genetic and environmental factors playing a role (Bhutto and Lutty, 2012). AMD can be divided into the dry, non-exudative form with cellular debris (drusen) accumulating between the Bruch’s membrane and the RPE layer, leading to an undersupply and atrophy of RPE cells, and a wet, exudative form with growing blood vessels behind the retina causing hemorrhaging. Appropriate *in vivo* models for this multifactorial eye disease are limited. Various animal models were created in mice, rats, or rabbits and resembled some of the histological features of AMD, but none of these models summarizes all of the conditions of human AMD (Zeiss, 2010; Pennesi et al., 2012b; Armento et al., 2020). Most models rely on damages like laser-induced injuries to the RPE and Bruch’s membrane or transgenic animals displaying AMD-like defects (Shah et al., 2015; Carver et al., 2017; Park et al., 2017; Tode et al., 2018). Furthermore, severe differences in immune response between humans and rodents make research of diseases that have a high inflammatory component challenging (Mestas and Hughes, 2004). The absence of a macula in these animals makes it even more difficult. Therefore, it can be concluded that all these models give only simplified information on the pathogenesis of this complex disease, as reviewed in detail in Schnichels et al. (2020). Due to the lack of suitable *ex vivo* models for AMD, so far, most studies on primary RPE cultures have been performed (Schnichels et al., 2020).

In contrast to commonly used retinal explants, which consist only of the detached retina, co-cultivation of primary RPE cells, human ARPE-19, or hTERT-RPE1 cells with neuroretina explanate is particularly important to create a functioning model for studying AMD pathogenesis (Armento et al., 2020; Wagner et al., 2020). Retinal explant cultures with RPE can be maintained *in vitro* for several weeks under entirely controlled conditions (Söderpalm et al., 1994). Choroid/RPE cultures are another important organ model in this research field and were used to study AMD-related molecular pathways. Klettner et al. (2013) demonstrated in RPE/choroid cultures that VEGF can be regulated independently by p38 and NFκB.

### Glaucoma

Glaucoma is defined by a loss of RGCs and optic nerve degeneration, leading to visual field defects. Since an elevated intraocular pressure (IOP) is the main risk factor, many glaucoma (animal) models rely on increased IOP or optic nerve damage (Bouhenni et al., 2012; Ishikawa et al., 2015; McMonnies, 2017). Glaucoma research mainly depends on rat and mouse models due to improved methods of inducing and evaluating glaucoma damage and the availability of genetic tools in rodents (Pang and Clark, 2020). In most of these models, the IOP is surgically increased by laser-induced damage or microbead injection (Zhang et al., 2017; Calkins et al., 2018; de Hoz et al., 2018). Other glaucoma models are optic nerve crush or axotomy (Schnichels et al., 2012b; Donahue et al., 2019). Although acute optic nerve damage models are often not suitable to mimic chronic glaucoma, they are useful to evaluate RGC pathogenesis and neuroregeneration (Nadal-Nicolás et al., 2017). Consequently, several retinal *ex vivo* models are based on physically or chemically induced damage of RGCs. Although even the axotomy leads to RGC injury, chemical treatment with hydrogen peroxide (H_2_O_2_) or cobalt chloride (CoCl_2_) may additionally induce oxidative stress or hypoxia-like processes in retinal cells (del Olmo-Aguado et al., 2013; Kuehn et al., 2017; Maliha et al., 2019). Incubation with H_2_O_2_ led to severe degeneration of RGCs with persistent apoptosis. Furthermore, a strong microglial response was observed, which was demonstrated by an increased expression of corresponding markers heat shock protein (HSP)70, inducible nitric oxide synthase (iNOS), and interleukin (IL)-1β (Hurst et al., 2017a). CoCl_2_ application on porcine retinal cultures resulted in a loss of RGCs as well as amacrine and bipolar cells. Interestingly, CoCl_2_ inhibited not only the number but also the activity of microglial cells (Kuehn et al., 2017). An effect of macroglia was not observed under any substance (Hurst et al., 2017a; Kuehn et al., 2017). Using these models, it was possible to perform therapy tests in which the neuroprotective effect of hypothermia and of the iNOS inhibitor 1400W on the different retinal cells was tested. It was found that both therapies provide improved RGC survival (Hurst et al., 2019, 2020; Maliha et al., 2019).

### Retinitis Pigmentosa

RP is a genetically determined multifactorial disease that leads to dystrophy of the photoreceptors (Hamel, 2006). Many animal models of (natural and transgenic) RP are available like rd1- and rd10 mice or S334-ter line rats and have led to a better understanding of the pathology of the disease and the development of therapeutic strategies (Pennesi et al., 2012a; Seiler et al., 2014). In case of the rd1 mice, a nonsense mutation in exon 7 of the Pde6b gene leads to the degeneration of rod photoreceptor within the first 3 weeks (Chang and Min, 2011). Therefore, the possible culture time for organ explants is perfect to investigate degenerative processes. Retinal postnatal day 2 (P2) explants from wild-type mice, as well as from rd1-mice (P2), can be cultivated for up to 4 weeks and follow the same developmental time course as that in *in vivo* littermates (Ogilvie et al., 1999). The cultured retinae follow the same developmental time course as that *in vivo* and provide the degeneration environment needed to study several factors and therapeutic treatments. A great advantage of these *ex vivo* cultures is that therapeutic treatments can be easily applied, whereas injection into the small eyes of young mice is challenging. Further, organ cultures enable a constant and controlled treatment for the wished duration (Ogilvie et al., 1999; Sahaboglu et al., 2010).

### Central Retinal Occlusion

CRAO is an acute, painless sudden occlusion of the central retinal artery, which first leads to ischemia and then vision loss (Graefe, 1859). The molecular mechanisms and reactions following retinal ischemia are complex, which makes the pathophysiological assessment very difficult (Osborne et al., 2004). Different models were developed for ischemia by either placing the organ cultures in a hypoxia chamber or by chemically inducing ischemia or neurodegeneration by adding glutamate. In the hypoxia *ex vivo* models, the retina is placed in culture, with the retina lying on a lower insert, i.e., the outer retina is supplied with oxygen and nutrients by the underlying medium. Hypoxia therefore mainly affects the inner retina. Thus, these *ex vivo* models are comparable to the *in vivo* situation where the outer retina is still nourished while the inner retina lacks oxygen, glucose, and other nutrients. These models were also used to test possible therapies as hypothermia, which represents an already established therapy in neurology (Schultheiss et al., 2016; Klemm et al., 2019). Hypothermia treatment on retinae under hypoxic stress prevented the death of RGCs and attenuated the gliosis response (Klemm et al., 2019). Testing this rather simple therapy *in vivo* would have been much more difficult or even impossible.

## Discussion

The culture system most similar to *in vivo* conditions is the retinal explant culture, which conserves the complex connections of neuronal and other functionally important non-neuronal cells of the retina. The presence of several cell types allows the study of *in vivo* dynamics of cell–cell interaction, immune responses, and degeneration pathways. Retinal explant models provide insights into retinal pathologies and can be used for therapy testing (Li et al., 2018; Cheng et al., 2019; Schnichels et al., 2020). However, their use in AMD research is still limited.

Yet, depending on the species used for organ culture, it needs to be assessed whether the results are at all transferable to the human situation. This transferability is not always possible for some organ cultures of rodents, but for organ cultures of higher mammals, it is quite realistic due to the high homology (Neitz and Neitz, 2001; Peichl, 2005; Volland et al., 2015; Murali et al., 2019; Schnichels et al., 2019). Further efforts to improve current *ex vivo* culture techniques should aim to produce models to fill this gap.

In summary, promising models are already developed, but a continuous further modification and refinement of these models are necessary to reflect the complexity of the retina more precisely.

## Author Contributions

JH, AF, and SS wrote and revised the manuscript. SJ and TT revised the manuscript. All authors read and approved the final version of the manuscript.

## Conflict of Interest

The authors declare that the research was conducted in the absence of any commercial or financial relationships that could be construed as a potential conflict of interest.
